# S100A16-induced adipogenesis is associated with up-regulation of 11 β-hydroxysteroid dehydrogenase type 1 (11β-HSD1)

**DOI:** 10.1042/BSR20182042

**Published:** 2019-09-09

**Authors:** Rihua Zhang, Jing Bao Kan, Shan Lu, Pei Tong, Jie Yang, Ling Xi, Xiubing Liang, Dongming Su, Dong Li, Yun Liu

**Affiliations:** 1 Department of Geratology, The First Affiliated Hospital of Nanjing Medical University, Nanjing 210029, China; 2 The Center of Metabolic Disease Research, Nanjing Medical University, Nanjing 210029, China; 3 Department of Orthopedics, Jiangsu Province Hospital of Chinese Medicine, Affiliated Hospital of Nanjing University of Chinese Medicine, Nanjing, Jiangsu, China

**Keywords:** 11β-HSD1, adipogenesis, obesity, S100A16, type 2 diabetes

## Abstract

The steadily increasing epidemic of obesity continues at alarming rates, is an important public health problem, and expression changes of S100A16 and 11 β-hydroxysteroid dehydrogenase type 1(11β-HSD1) is attributable to the adipocyte differentiation. In our previous study, we found that 11β-HSD1 protein expression increased in S100A16-overexpressed 3T3-L1 cell model. In order to further investigate the relationship between S100A16 and 11β-HSD1, and the molecular mechanisms of S100A16-induced adipogenesis, we constructed S100A16 transgenic and knockout mouse, and S100A16-overexpressed 3T3-L1 preadipocyte cell. Using S100A16 transgenic (S100A16^Tg/+^) mice fed with normal fat diet (NFD) and high fat diet (HFD) diet model, we evaluated the effect of S100A16 on adipogenesis, expression of 11β-HSD1, and RNA sequencing and quantification of gene expression. Using the 3T3-L1 cell model, we examined the effect of S100A16 and 11β-HSD1 on pre-adipocyte differentiation, and cell signaling events of 11β-HSD1 overexpression induced by S100A16. We found that when compared with C57BL/6 mice, overexpression of S100A16 under the condition of HFD increased lipid content in WAT and fat infiltration in hepatocytes, 11β-HSD1 protein expression increased along with S100A16. Elevated S100A16 and 11β-HSD1 expression promoted adipogenesis in 3T3-L1 cells. Overexpression of S100A16 inhibited the degradation of 11β-HSD1. We conclude that S100A16-induced adipogenesis is associated with up-regulation of 11β-HSD1.

## Introduction

Obesity is an important metabolic disorder and serious public health problem worldwide that is closely associated with various dangerous disease risk factors, such as insulin resistance and Type 2 diabetes. Due to the wide-ranging health implications, the need to better understand the cellular and molecular basis of adipocyte differentiation in order to develop new and effective strategies for obesity prevention has become acute [1,2]. During adipocyte differentiation, chronological changes in the expression of numerous genes take place throughout the process, including C/EBP-α, PPAR-γ, S100A16, and 11β-HSD1 [3–5].

S100 proteins are small, acidic calcium-binding proteins of 10–12 kDa in size that contain two distinct EF-hand motifs. The S100-specific EF-hand is located at the N-terminus, followed by a classical Ca^2+^-binding EF-hand that operates as a Ca^2+^-activated switch that interacts with and modulates the activity of a large number of targets [6]. The S100 protein family is involved in the regulation of diverse cellular processes such as cell growth, differentiation, and cell cycle progression. The recently identified S100 protein family member S100A16 has been linked with obesity, Type 2 diabetes, inflammation, and cancer, via a Ca^2+^-dependent mechanism [4,7–12]. S100A16 is expressed widely in human tissues, and its precise biological functions are not fully understood.

The enzyme hydroxysteroid dehydrogenase type 1 (11β-HSD1) is a key enzyme that catalyzes the intracellular conversion of cortisone to physiologically active cortisol [13,14], and this enzyme has received widespread attention in the past decade. A series of studies showed that 11β-HSD1 can promote preadipocyte differentiation and adipogenesis, and cause insulin resistance [15,16]. Additionally, overexpression of 11β-HSD1 in adipose tissue is a key feature of metabolic syndrome [17,18].

In our present study, we demonstrated that up-regulation of S100A16 expression in adipose tissue promoted 11β-HSD1 expression, while down-regulation suppressed 11β-HSD1 expression, suggesting S100A16 may be associated with pre-adipocyte differentiation, obesity, and insulin resistance. We used an S100A16 transgenic (S100A16^Tg/+^) mouse model fed with a high fat diet (HFD) to investigate the effect of S100A16 on adipogenesis and the expression of 11β-HSD1. Cultured adipocytes were used to investigate the effect of S100A16 and 11β-HSD1 on preadipocyte differentiation, and the molecular mechanism of up-regulation of 11β-HSD1 induced by overexpression of S100A16 was explored.

## Materials and methods

### Mouse breeding and genotyping

S100A16 transgenic heterozygous mouse (S100A16^Tg/+^) and S100A16 knockout heterozygous mouse (S100A16^KO/+^) were generated at the Model Animal Research Center Of Nanjing University (Contract [2008] 054, [2009] T 67). The promoter used for S100A16^Tg/+^ mouse was CAG. The genetic background of S100A16^Tg/+^ and S100A16^KO/+^ was C57BL/6. All mice were housed, fed, and monitored at the Model Animal Research Center of Nanjing University (MARC), with 12-h light/dark cycles and with free access to food and water. The facility is maintained in a constant temperature and humidity environmental conditions. The Ethics Committees, the Institutional Animal Care and Use Committee (IACUC) at the University of Nanjing Medical University, approved all animal protocols. Genotyping was carried out using primers listed in Supplementary Figures S1 and S3B. Unfortunately, we were unable to obtain sufficient heterozygous S100A16^KO/+^ mice, presumably because knockdown of S100A16 may have affected their reproductive capacity. We cannot get homozygous S100A16^KO/ KO^ mouse. And this is being investigated separately within our group.

A total of 10 C57BL/6 and 10 S100A16^Tg/+^ 5-week-old male mice were fed with a NFD or HFD diet for 17 weeks (from 5 to 21 weeks of age). The HFD was supplied by OpenSource DIETS (Research Diets, Inc., New Brunswick, NJ, U.S.A., #D12451, the details of the NFD is as Supplementary Table S1). We monitored the body weight of all mice every week and measured the visceral fat weight following administration of the anesthetic Nembutal (100 mg/kg). In addition, we investigated the morphology of visceral fat cells from mice fed normal chow and a HFD using hematoxylin and eosin staining.

### Cell culture and differentiation

The 3T3-L1 mouse preadipocyte cell line was purchased from ATCC and cultured in high glucose Dulbecco’s modified Eagle medium (DMEM; Thermo Fisher) containing 10% newborn calf serum, 100 U/ml penicillin, and 100 mg/ml streptomycin in 5% CO_2_ at 37°C. Preadipocyte differentiation was induced as described previously [19]. To induce adipocyte differentiation, 3T3-L1 cells were seeded and allowed to grow for 2 days to reach confluence (designated as day 0), then cultured with DMEM containing 10% FBS and 0.5 mM 3-isobutyl-1-methyxanthine, 1 μg/ml porcine insulin, and 1 mM dexamethasone (MIX, Sigma). After 48 h of incubation (designated as day 2), the medium was replaced with DMEM containing 10% FBS and 1 μg/ml insulin. On day 4, cells were cultured with DMEM containing 10% FBS and the incubation was continued for 4 days with two changes of medium.

### Construction of S100A16 and 11β-HSD1 interference expression plasmids, transfection, and stable clone selection

Four distinct domains within the coding region of mouse S100A16 (NM_026416.2) and 11β-HSD1 (NM_001044751) cDNAs were targeted for RNA interference. For this purpose, four pairs of reverse complementary oligonucleotides targeting mouse S100A16 were designed and sequences are given in Supplementary Table S2. Selected regions of 11β-HSD1 were also targeted by siRNA using the primers of Supplementary Table S3. Briefly, total RNA was purified using Trizol Reagent (Invitrogen).

Oligonucleotides were annealed and inserted into the pcDNA6.2-GW/EmGFP-miR expression vector (Invitrogen, #K4936-00). And then we generated pcDNA6.2-GW/EmGFP-miR-S100A16-1, 2, 3, and 4, and pcDNA6.2-GW/EmGFP-miR-11β-HSD1-1, 2, 3, and 4. A control construct was also created.

We used X-tremeGENE HP DNA Transfection Reagent (Roche, (#06365752001) to separately transfect the nine different plasmids into 3T3-L1 cells. To select for successful transfectants, cells were cultured at 24 h after transfection in selection medium containing 4 μg/ml blasticidin (Sigma). Blasticidin-resistant cells were maintained in culture medium supplemented with 2 μg/ml blasticidin for further analysis.

### Construction of S100A16 and 11β-HSD1 overexpression plasmids, transfection and stable clone selection

Overexpression plasmid pcDNA3.1(+)-S100A16 and pcDNA3.1(+)-11β-HSD1 were constructed by synthesizing full-length mouse S100A16 (NM_026416.2) and 11β-HSD1 (NM_001044751) cDNAs and cloning into pcDNA3.1(+) using *Hind*III and *EcoR*I restriction sites. Plasmid DNA was purified from transformed bacteria and the final construct was verified by sequencing. The sequence congruence within the used restriction sites was 100%.

Expression constructs were transfected into 3T3-L1 preadipocytes using X-tremeGENE HP DNA Transfection Reagent (Roche, #06365752001). After 48 h, cells were cultured in selective DMEM medium containing 600 μg/ml G418 (Sigma) for 2 weeks for the selection of resistant colonies. G418-resistant cells were maintained in culture medium supplemented with 300 μg/ml G418 for further analysis.

### Oil Red O staining

Oil Red O (O0625) was purchased from Sigma-Aldrich (St. Louis, MO). Differentiated 3T3-L1 cells (day 0, 4, or 10) were washed three times with PBS and stained with filtered Oil Red O solution (stock solution, 3 mg/ml in isopropanol; working solution, 60% stock solution and 40% distilled water) for 60 min at room temperature. Cells were then washed with ddH_2_O to remove unbound dye, and visualized and photographed under a microscope.

### Protein extraction and Western blotting analysis

Tissues and cells were washed twice with ice-cold PBS, and 100 mg tissue was lysed with 1 ml lysis buffer (50 mM TRIS-HCl pH 7.4, 150 mM NaCl, 1% v/v Nonidet-P40, 1 mM EDTA, 1 mM NaF, 10 mg/ml aprotinin, 10 mM leupeptin, and 1 mM phenylmethanesulfonyl fluoride). Cells were scraped into lysis buffer, and tissues and cells were incubated on ice for 30 min. After centrifugation at 4°C, proteins in the supernatant were extracted, and the concentration was detected using BCA Protein Assay Kit (23225, PIERCE, U.S.A.). The protein was separated by SDS-PAGE then subjected to a standard Western blotting assay and imaged using a Molecular Imager ChemiDoc XRSC with the Image Lab Software (Version 4.0.1, Bio-Rad Laboratories, Hercules, CA, U.S.A.). Antibodies for 11β-HSD1 (AF3397) were purchased from R&D SYSTEMS, α-Tubulin (T5168) antibody was purchased from Sigma-Aldrich (St. Louis, MO), and S100A16 antibody (ab130419) was purchased from Abcam (U.S.A.).

### Intraperitoneal glucose tolerance test (IGTT) and insulin tolerance test (ITT)

After overnight fasting, the tail blood glucose concentration (mM) was recorded (designated time = 0 min), mice were injected intraperitoneally with glucose at a dose of 2 g/kg of body weight, and blood glucose levels were monitored using a handheld glucometer (ACCU-CHEK Performa, Roche) at 15, 30, 60, and 120 min.

For ITTs, all mice were starved for 12 h, injected with a bolus of insulin (0.3 unit/kg of body weight), and their blood glucose levels were monitored using a handheld glucometer (ACCU-CHEK Performa, Roche) at 15, 30, 60, and 120 min.

### RNA sequencing and quantification of gene expression

Adipose tissues from C57BL/6, S100A16^KO/+^ and S100A16^Tg/+^ (*n* = 3) mice were used for total RNA extraction using TRIzol reagent (Invitrogen), and RNA quality was tested using a Bioanalyzer 2200 (Agilent) instrument, samples with RIN ≥ 8.0 were used for RNA sequencing by BGI Tech Solutions. Briefly, total RNA was treated with Dnase I, mRNA was enriched using oligo(dT) magnetic beads. Then, the mRNA fragments is used for synthesis of the first and second strands of cDNA. The double-stranded cDNA products were sequenced using a Illumina HiSeq 2000. The RNA seq data analysis was also performed by BGI Tech Solutions.

### Mouse embryonic fibroblasts (MEFs)

MEFs were isolated from Wild-type (C57BL/6), S100A16^Tg/Tg^ and S100A16^KO/+^ mouse embryos at 13.5 days post coitum. Briefly, embryos were chopped into pieces and incubated in 0.025% trypsin and 0.5 mM EDTA at 37°C for 60 min with periodic agitation. Cells were washed with DMEM containing 10% FBS and dispersed by pipeting. S100A16 protein levels were determined using Western blotting. Cells were treated with 20 μM cycloheximide (CHX; C7698, Sigma) for 0, 12, or 24 h, and 11β-HSD1 protein expression was analyzed by Western blotting.

### Triglyceride GPO-POD assay

Cellular triglyceride content was determined by using a Triglyceride GPO-POD Assay Kit (Sigma) according to a previously published method [4]. 3T3-L1 cells were cultured and induced in 10-cm well to differentiate into adipocytes (0 d, 4 d, and 10 d) before being washed with PBS twice, scraped in 500 μl PBS, sonicated to homogenize the suspension, and then assayed for total triglyceride.

### Statistical analysis

Results are expressed as the mean ± SD. Data from two groups were compared using unpaired Student’s *t* tests. A *P* value less than 0.05 was considered statistically significant.

## Results

### Overexpression of S100A16 causes insulin resistance and lipid droplet accumulation in mice

Genotyping of S100A16 transgenic (S100A16^Tg/+^) mice was performed using standard PCR screening of tail genomic DNA with specific primers (Supplementary Figure S1). The tissue specificity of the transgenic at mRNA levels was determined using Q-PCR (Supplementary Figure S2). The transgene was expressed at high levels in all tissues, and expression was especially high in white adipose tissue (WAT) and liver. S100A16^KO/+^ was generated for use as a negative control; however, reproductive capacity was limited in these animals and prevented further use as a control. Investigations into the reproductive ability of the animals are continuing in our laboratory. Genotyping of S100A16^KO/+^ mice was performed using standard PCR screening of tail genomic DNA with specific primers (Supplementary Figure S3).

**Figure 1:**
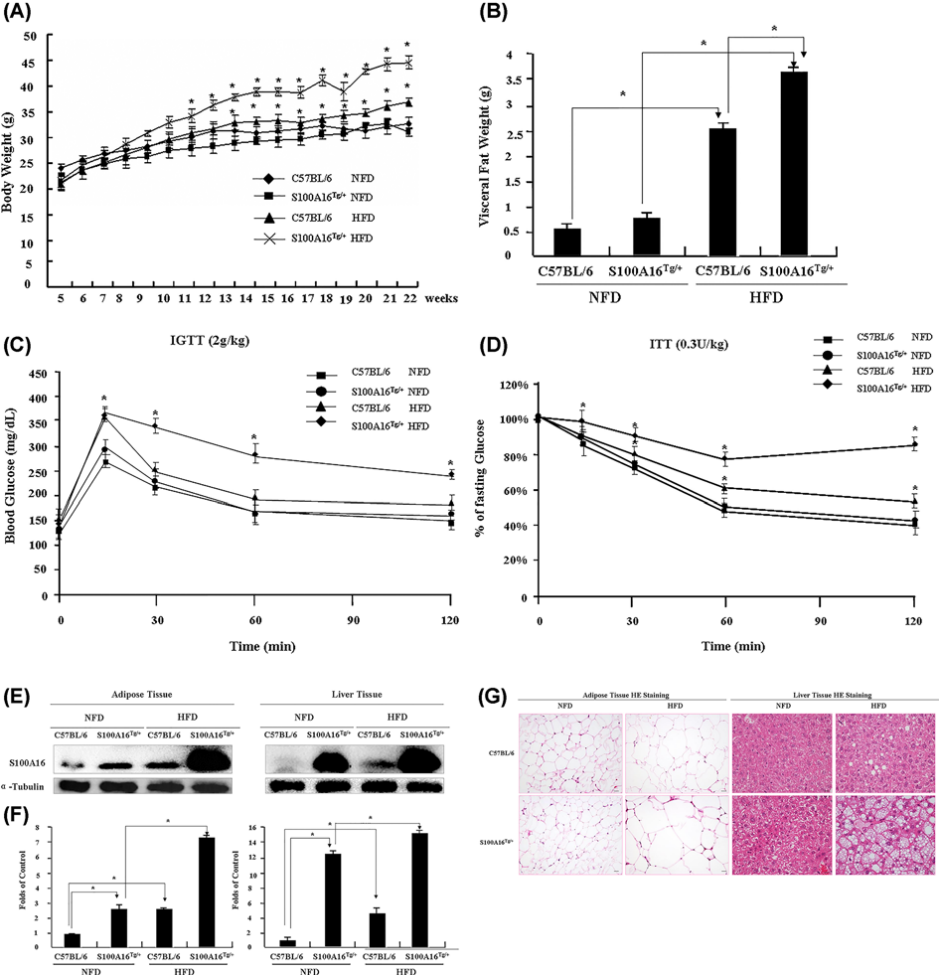
Effects of S100A16 on fat metabolism and insulin resistance C57BL/6 and S100A16^Tg/+^ male mice were fed a NFD or HFD from 5 to 21 weeks of age (*n* = 6 for C57BL/6 NFD mice, *n* = 5 for S100A16^Tg/+^ NFD mice, *n* = 14 for C57BL/6 HFD mice, *n* = 8 for S100A16^Tg/+^ HFD mice). (**A**) Body weight monitored every week. (**B**) Visceral fat weight measured following administration of the anesthetic Nembutal (100 mg/kg). (**C**) At 14 and 20 weeks, intraperitoneal glucose tolerance tests (IGTT) were performed twice. All mice were starved overnight, then injected intraperitoneally with glucose at a dose of 2 g/kg of body weight, and blood glucose levels were monitored using a handheld glucometer at 15, 30, 60, and 120 min. (**D**) The response in blood glucose following an intraperitoneal injection of insulin (0.3 unit/kg of body weight). At 15 and 21 weeks, insulin tolerance tests (ITT) were performed twice. All mice were starved for 12 h, then injected with a bolus of insulin (0.3 unit/kg of body weight), and blood glucose levels were monitored using the handheld glucometer at 15, 30, 60, and 120 min, and blood glucose concentrations at different time points were expressed as a percentage of the fasting blood glucose concentrations. (**E**) At the end of the study, adipose and liver tissues were removed immediately, and S100A16 protein levels were determined by a Western blotting with an S100A16-specific antibody. (**F**) Relative expression of S100A16 based on grayscale analysis with α-tubulin as a control. (**G**) Images of visceral fat cells and liver cells. Adipose and liver tissues were removed, immediately fixed with formalin and embedded in paraffin, and subjected to hematoxylin and eosin (HE) staining (bar = 20 μm).

To study the effect of S100A16 overexpression on fat and blood glucose metabolism, the mice (C57BL/6 and S100A16^Tg/+^ mice) were fed with either a normal fat diet (NFD) or a high fat diet (HFD) for 17 weeks (from 5 to 21 weeks old). The bodyweight was measured every week, and S100A16^Tg/+^ HFD mice gradually developed a significantly higher body weight than S100A16^Tg/+^ NFD and C57BL/6 mice. The body weight of C57BL/6 HFD mice was also higher than C57BL/6 NFD mice. There was no difference between S100A16^Tg/+^ and C57BL/6 NFD groups (Figure 1A). At the experimental end point, the visceral fat was weighed, and visceral fat pad in the HFD groups consistently exceeded that of the NFD groups, with the highest visceral fat weight occurring in S100A16^Tg/+^ HFD mice (Figure 1B). To assess the impact of S100A16 on glucose metabolism, intraperitoneal glucose tolerance tests (IGTT) and insulin tolerance tests (ITT) were performed during the middle and at the end of feeding. In response to intraperitoneal (IP) glucose infusion (2 g/kg body weight), blood glucose levels were markedly elevated in different groups of mice, with levels in the HFD groups higher than those in NFD mice. However, the elevated blood glucose levels in NFD fed mice (both C57BL/6 and S100A16^Tg/+^) returned to normal within 120 min. Similarly, blood glucose levels in S100A16^Tg/+^ HFD mice also decreased, but did not return to normal within 120 min. S100A16^Tg/+^ mice in the HFD group displayed impaired glucose profiles (Figure 1C). Consistent with the observed glucose intolerance, the response of the blood glucose concentration to an IP injection of insulin was also impaired (Figure 1D). These results demonstrate that overexpression of S100A16 under HFD conditions can result in insulin resistance. After feeding, the expression of S100A16 was evaluated using Western blotting, and the results indicated that S100A16 was overexpressed in adipose tissue (3-fold) and liver tissue (13-fold), and HFD clearly induced S100A16 protein expression in fat tissue (Figure 1E,F). Histological analysis of WAT revealed that the sizes of adipocytes and the lipid droplet accumulation in hepatocytes in S100A16^Tg/+^ HFD mice were above those in wild-type mice and S100A16^Tg/+^ NFD mice (Figure 1G). These results demonstrated that overexpression of S100A16 under HFD conditions increased the lipid content in WAT and advanced the fat infiltration in hepatocytes.

### Effects of S100A16 on gene expression

To further explore the effect of S100A16 by molecular profiling, S100A16^Tg/Tg^ and S100A16^KO/+^ mouse adipose tissue transcriptomic analyses were performed using the RNA seq as described in the ‘Materials and Methods’ section. Analysis of differentially expressed genes between C57BL/6 and S100A16^Tg/Tg^ mice identified many genes that were up- or down-regulated at least 6-fold in S100A16^Tg/Tg^ adipose tissue (Table 1). Similarly, various genes expression were altered more than 5-fold between C57BL/6 and S100A16^KO/+^ mice (Table 2).

**Table 1:** Genes differentially expressed in the adipose tissue of C57BL/6 and S100A16^Tg/Tg^ mice

Gene ID	Symbol	log_2_ ratio (S100A16^Tg/Tg^/ C57BL/6)	*P*-value	FDR
84506	Hamp	9.523955165	5.74E-09	1.51E-08
12501	Cd3e	9.397419271	4.46E-27	2.32E-26
69496	Dydc1	9.360359767	1.55E-18	6.19E-18
114564	Csprs	9.359359448	2.75E-49	2.35E-48
231932	Gimap7	9.217173553	1.31E-21	5.77E-21
170786	Cd209a	9.212480404	1.10E-24	5.33E-24
328561	Apol10b	9.037862288	3.97E-29	2.18E-28
106648	Cyp4f15	8.986605799	1.71E-30	9.73E-30
433637	Gm5547	8.863585262	1.41E-06	3.23E-06
73652	2210408F21Rik	8.763337322	1.00E-12	3.16E-12
21935	Tnfrsf17	8.760491769	2.47E-10	6.98E-10
622523	Gm6329	8.567368423	1.19E-09	3.24E-09
667213	Gm10416	8.53997079	2.93E-07	6.99E-07
381484	Gm5150	8.484441432	1.19E-09	3.24E-09
93675	Clec2i	8.313961722	2.61E-09	7.00E-09
320679	Samd12	8.274755981	1.26E-08	3.26E-08
241452	Dhrs9	8.177416789	2.49E-81	3.49E-80
14990	H2-M2	8.170326953	1.00E-12	3.16E-12
93674	Cml3	8.166084423	4.06E-15	1.42E-14
72049	Tnfrsf13c	8.148405115	1.85E-15	6.54E-15
14080	Fabp1	8.135389877	0.000159	0.00031
381693	Wdr95	8.122545078	1.47E-19	6.06E-19
235712	Mrgpra2b	8.074152248	5.42E-10	1.50E-09
208154	Btla	8.072334743	1.49E-75	1.94E-74
631323	Gm12250	8.013441054	1.70E-72	2.12E-71
1.01E+08	1810008I18Rik	7.993285786	1.50E-05	3.18E-05
83558	Tex11	7.952248761	1.23E-22	5.62E-22
16429	Itln1	7.844627104	6.08E-08	1.51E-07
98256	Kmo	7.815705112	3.83E-16	1.40E-15
217306	Cd300e	7.808911324	1.00E-12	3.16E-12
238722	Zfp72	7.806682871	1.12E-10	3.23E-10
68400	0610043K17Rik	7.806441355	0.000159	0.00031
209380	Gm4759	7.805974539	1.47E-19	6.06E-19
67330	1700047M11Rik	7.781370499	7.23E-05	0.000146
414081	5330413P13Rik	7.769601473	2.71E-22	1.22E-21
227288	Cxcr1	7.730690992	2.93E-07	6.99E-07
236069	Gm13238	7.722722726	3.29E-05	6.80E-05
110895	Slc9a4	7.718360701	1.23E-22	5.62E-22
11604	Agrp	7.717141694	3.29E-05	6.79E-05
243958	Siglecg	7.697575311	8.92E-15	3.07E-14
16175	Il1a	7.67156649	1.06E-11	3.20E-11
20302	Ccl3	7.667860791	3.29E-05	6.79E-05
1.01E+08	Abhd12b	7.629120008	1.41E-06	3.23E-06
15018	H2-Q7	7.618472764	5.74E-09	1.51E-08
18507	Pax5	7.549993026	6.44E-07	1.50E-06
320026	A330076H08Rik	7.531009923	5.42E-10	1.50E-09
74748	Slamf8	7.531009923	5.11E-11	1.49E-10
17381	Mmp12	7.525340093	1.87E-51	1.66E-50
75526	Eppin	7.517571549	0.000159	0.00031
383787	Ankrd63	7.500718362	1.10E-24	5.33E-24
382045	Gpr114	7.478921412	1.96E-14	6.64E-14
76024	Gm11346	7.453565837	3.11E-06	6.93E-06
103098	Slc6a15	7.453270173	3.41E-18	1.34E-17
353208	Zfp931	7.40658447	5.74E-09	1.51E-08
72481	2610203C22Rik	7.40443941	1.41E-06	3.23E-06
791403	D830015G02Rik	7.384417425	0.000159	0.00031
22262	Uox	7.355594909	5.74E-09	1.51E-08
15930	Ido1	7.337701994	1.34E-07	3.25E-07
16631	Klra13-ps	7.26974461	0.000349	0.000662
70086	Cysltr2	7.262521087	2.77E-08	7.02E-08
21949	Tnfsf8	7.251826749	5.42E-10	1.50E-09
15551	Htr1b	7.244675429	1.50E-05	3.18E-05
11889	Asgr1	7.243433333	6.82E-06	1.48E-05
637515	Nlrp1b	7.240307984	7.50E-18	2.91E-17
223917	Krt79	7.235833476	5.66E-42	4.22E-41
76074	Gbp8	7.201068057	5.86E-41	4.27E-40
1.01E+08	C230037L18Rik	7.172216534	0.000349	0.000662
78849	B430010I23Rik	7.171442888	3.63E-79	4.96E-78
68236	Gtsf1l	7.159041146	0.000349	0.000662
70489	5730405O15Rik	7.147610565	0.000349	0.000662
12994	Csn3	7.147249168	6.44E-07	1.50E-06
239081	Tlr11	7.139141588	4.56E-13	1.46E-12
21947	Cd40lg	7.138115307	1.50E-05	3.18E-05
232408	Klrb1f	7.128308018	2.93E-07	6.99E-07
667736	Gm8787	7.084494192	6.82E-06	1.48E-05
668108	Gm8979	7.078732515	1.41E-37	9.54E-37
140491	Ppp1r3a	7.078732515	4.27E-74	5.44E-73
232406	BC035044	7.074888505	2.47E-10	6.98E-10
94222	Olig3	7.05138094	2.77E-08	7.02E-08
1.01E+08	4930469G21Rik	7.042555403	0.000349	0.000662
69191	Pdia2	7.039366384	6.44E-07	1.50E-06
11997	Akr1b7	7.033510263	3.29E-05	6.79E-05
11871	Art2a-ps	7.023143548	3.29E-05	6.79E-05
66773	Gm17019	7.009909904	7.23E-05	0.000146
381287	A530032D15Rik	7.007038052	6.44E-07	1.50E-06
244550	Podnl1	6.988422374	6.08E-08	1.51E-07
18231	Nxph1	6.988306778	6.44E-07	1.50E-06
14762	Gpr33	6.971528393	1.50E-05	3.18E-05
72958	Zfp493	6.941634158	1.06E-11	3.20E-11
236727	Slc9a7	6.933724144	5.74E-09	1.51E-08
67717	Lipf	6.916966358	3.29E-05	6.79E-05
18788	Serpinb2	6.895575376	2.93E-07	6.99E-07
58175	Rgs20	6.887153733	1.34E-07	3.25E-07
1.01E+08	AW046200	6.877005778	1.50E-05	3.18E-05
11835	Ar	6.875561879	2.47E-10	6.98E-10
210757	Themis	6.869010974	2.07E-13	6.74E-13
20750	Spp1	6.836426628	0	0
382864	Colq	6.813142404	3.66E-31	2.12E-30
653030	Arhgap27os3	6.788221666	5.74E-09	1.51E-08
53315	Sult1d1	6.787333217	1.22E-89	1.89E-88
442820	D830005E20Rik	6.769849824	3.11E-06	6.93E-06
18171	Nr1i2	6.767291601	2.77E-08	7.02E-08
54167	Icos	6.766348192	3.75E-30	2.13E-29
13586	Ear1	6.766348192	3.75E-30	2.12E-29
12775	Ccr7	6.734286983	1.77E-29	9.82E-29
1E+08	Cyp4a32	6.71609765	3.11E-06	6.93E-06
23960	Oas1g	6.657746769	3.11E-06	6.93E-06
13590	Lefty1	6.655354378	3.29E-05	6.80E-05
242248	Bank1	6.633592983	1.86E-27	9.76E-27
73895	4930431P03Rik	6.632314689	6.44E-07	1.50E-06
269346	Slc28a2	6.616105557	4.03E-27	2.10E-26
320046	F730043M19Rik	6.604452294	7.23E-05	0.000146
226695	Ifi205	6.580481647	1.90E-26	9.72E-26
1.01E+08	9230112J17Rik	6.531009923	6.44E-07	1.50E-06
620078	C130026I21Rik	6.519081102	8.10E-74	1.03E-72
433809	Rnf207	6.51285521	6.44E-07	1.50E-06
14103	Fasl	6.507709835	1.50E-05	3.18E-05
19419	Rasgrp1	6.506481066	3.77E-49	3.21E-48
16519	Kcnj3	6.49004875	7.23E-05	0.000146
78016	Ccdc150	6.487372243	9.12E-25	4.43E-24
22445	Xlr3a	6.477217106	7.23E-05	0.000146
268934	Grm4	6.459081925	6.08E-08	1.51E-07
216864	Mgl2	6.448378111	3.91E-47	3.22E-46
27278	Clnk	6.446727327	0.000159	0.00031
12902	Cr2	6.445057088	1.85E-15	6.55E-15
15560	Htr2c	6.444606385	4.83E-12	1.48E-11
11498	Adam4	6.432358525	3.11E-06	6.93E-06
108956	Apol7c	6.428648205	1.50E-05	3.19E-05
209590	Il23r	6.425164835	1.41E-06	3.23E-06
21822	Tgtp1	6.408300672	2.01E-23	9.41E-23
574437	Xlr3b	6.40194735	0.000159	0.00031
68404	Nrn1	6.401511506	9.00E-68	1.04E-66
636104	Gm7173	6.398794728	2.93E-07	6.99E-07
20558	Slfn4	6.391267474	0	0
382074	Foxr1	6.375731697	0.000159	0.00031
1E+08	Gm14548	6.369189971	1.50E-05	3.18E-05
244723	Olfm2	6.365807332	0.000349	0.000661
14598	Ggt1	6.346016393	2.05E-22	9.23E-22
80901	Cxcr6	6.346016393	2.05E-22	9.23E-22
15950	Ifi203	6.32032975	0	0
234673	Ces2e	6.307726344	1.41E-06	3.23E-06
217122	Gm11545	6.303456433	1.41E-06	3.23E-06
280667	Adam1b	6.294542316	6.44E-07	1.50E-06
1.01E+08	Gm13293	6.287555886	0.000349	0.000661
70928	Trim69	6.279432582	0.000159	0.00031
14562	Gdf3	6.276569103	3.29E-05	6.80E-05
319940	Sorbs2os	6.252733575	6.82E-06	1.48E-05
27274	Zfp354b	6.24111761	6.82E-06	1.48E-05
20981	Syt3	6.223867165	6.82E-06	1.48E-05
243659	Styk1	6.218447246	1.34E-07	3.25E-07
227485	Cdh19	6.211913419	6.44E-07	1.50E-06
226565	Fmo6	6.209081828	0.000159	0.00031
399570	Kank4os	6.204240565	1.50E-05	3.18E-05
13086	Cyp2a4	6.196407998	0.000349	0.000662
23845	Clec5a	6.18929089	4.37E-39	3.07E-38
1E+08	Dthd1	6.174937151	1.50E-05	3.18E-05
320692	9430037G07Rik	6.154948391	6.44E-07	1.50E-06
17472	Gbp4	6.147617151	0	0
667214	9930111J21Rik1	6.141196601	1.40E-92	2.26E-91
239789	Gmnc	6.137463105	1.26E-08	3.26E-08
170745	Xpnpep2	6.116534547	4.59E-19	1.86E-18
320558	Sycp2	6.116534547	4.59E-19	1.86E-18
57765	Tbx21	6.108415072	1.50E-05	3.19E-05
244233	Cd163l1	6.104043603	9.61E-37	6.38E-36
320484	Rasal3	6.091443566	2.08E-36	1.37E-35
236312	Pyhin1	6.091443566	4.89E-54	4.56E-53
225825	Cd226	6.065908474	9.67E-36	6.28E-35
328563	Apol11b	6.065908474	2.14E-18	8.49E-18
210108	D130043K22Rik	6.039913266	4.62E-18	1.81E-17
20299	Ccl22	6.039913266	4.62E-18	1.81E-17
380842	Stmnd1	6.013441054	1.23E-67	1.42E-66
14525	Gcsam	6.013441054	9.98E-18	3.85E-17
60504	Il21r	6.013441054	9.98E-18	3.85E-17
13386	Dlk1	-15.12358461	0	0
14955	H19	-10.69854849	0	0
21952	Tnni1	-10.09528979	1.67E-30	9.50E-30
15126	Hba-x	-8.704567383	2.76E-07	6.59E-07
57255	Cldn13	-8.250720441	7.21E-09	1.89E-08
12824	Col2a1	-8.249011178	5.28E-40	3.77E-39
27206	Nrk	-8.202176268	1.67E-49	1.43E-48
11609	Agtr2	-8.120006692	9.64E-21	4.13E-20
23934	Ly6h	-7.969819615	7.21E-09	1.89E-08
228770	Rspo4	-7.955994187	1.83E-15	6.47E-15
75740	Egfem1	-7.82450975	5.41E-16	1.96E-15
11606	Agt	-7.823595489	0	0
80982	Cemip	-7.72334529	2.02E-38	1.40E-37
20197	S100a3	-7.584625499	0.000405	0.000763
14371	Fzd9	-7.40435477	1.16E-09	3.18E-09
232345	A2m	-7.355294691	5.97E-20	2.50E-19
14840	Gsg1	-7.18200142	5.76E-06	1.26E-05
16939	Lor	-7.070501697	9.30E-07	2.15E-06
70281	2310068J16Rik	-7.036703571	0.00022	0.000425
22776	Zim1	-6.979851187	1.10E-38	7.67E-38
14621	Gjb4	-6.950528276	3.56E-05	7.33E-05
233332	Adamts17	-6.867290381	0	0
13717	Eln	-6.851388429	0	0
16682	Krt4	-6.815374692	9.30E-07	2.15E-06
22044	Trh	-6.763814261	3.30E-33	2.01E-32
619665	Klf14	-6.631026918	4.46E-08	1.11E-07
71775	1300017J02Rik	-6.530196477	5.76E-06	1.26E-05
230587	Glis1	-6.441055975	5.07E-07	1.19E-06
280635	Emilin3	-6.310272311	8.19E-08	2.01E-07
209966	Pgbd5	-6.288340908	1.87E-46	1.53E-45
14603	Gif	-6.225262225	2.22E-44	1.74E-43
215303	Camk1g	-6.222573138	6.69E-66	7.52E-65
140494	Atp6v0a4	-6.205691868	0	0
12291	Cacna1g	-6.133763871	1.57E-41	1.15E-40
626009	Gm6644	-6.098998453	9.77E-21	4.19E-20
17885	Myh8	-6.095711215	3.04E-11	8.94E-11
29873	Cspg5	-6.081562684	2.76E-07	6.59E-07
12839	Col9a1	-6.020069209	5.07E-07	1.19E-06

**Table 2:** Genes differentially expressed in the adipose tissue of C57BL/6 and S100A16^KO/+^ mice

Gene ID	Symbol	log2 ratio (S100A16^KO/+^/57BL/6)	*P*-value	FDR
20302	Ccl3	9.775108	4.69E-21	3.17E-20
84506	Hamp	9.161717	1.69E-07	5.63E-07
14080	Fabp1	8.462584	9.91E-06	2.85E-05
1E+08	Gm17455	8.356806	5.68E-09	2.09E-08
20700	Serpina1a	8.151426	1.24E-16	6.88E-16
103149	Upb1	7.281212	3.33E-07	1.08E-06
218763	Lrrc3b	7.250685	4.35E-08	1.51E-07
12346	Car1	7.093634	1.95E-05	5.46E-05
11699	Ambp	7.049678	1.95E-05	5.46E-05
22262	Uox	6.63972	5.03E-06	1.48E-05
67749	Mgarp	6.629135	7.59E-05	0.000199
75986	Agmat	6.447271	0.000295	0.000722
268756	Gulo	6.259803	9.91E-06	2.85E-05
19850	Rnu3a	6.127641	2.26E-20	1.49E-19
414081	5330413P13Rik	6.076447	8.57E-08	2.91E-07
319616	5930412G12Rik	6.045179	3.24E-19	2.03E-18
12116	Bhmt	5.995014	0.00015	0.00038
223780	Adm2	5.972666	1.05E-52	1.59E-51
243958	Siglecg	5.739626	0.00015	0.00038
11287	Pzp	5.712604	3.47E-15	1.81E-14
78250	Iqch	5.596964	1.95E-05	5.46E-05
110135	Fgb	5.527572	1.20E-25	9.41E-25
69121	Chrdl2	5.383981	4.89E-12	2.18E-11
14161	Fga	5.350034	1.66E-22	1.17E-21
72958	Zfp493	5.225759	0.000295	0.000722
20704	Serpina1e	5.036667	6.12E-43	7.61E-42
381813	Prmt8	-7.73802	8.49E-16	4.53E-15
99738	Kcnc4	-7.19525	1.21E-13	5.81E-13
22044	Trh	-7.06465	7.17E-39	8.11E-38
52793	Fam3b	-7.05282	0.000409	0.000984
13076	Cyp1a1	-6.70754	4.93E-09	1.82E-08
58251	BC100451	-6.48666	5.85E-06	1.72E-05
18741	Pitx2	-6.43479	1.42E-06	4.37E-06
20608	Sstr4	-6.38056	0.000409	0.000984
238393	Serpina3f	-6.30911	5.85E-06	1.72E-05
14748	Gpr3	-6.28377	1.19E-05	3.39E-05
17883	Myh3	-6.27053	2.93E-14	1.45E-13
69852	Tcf23	-6.26505	5.89E-10	2.32E-09
96875	Prg4	-6.10837	2.96E-57	4.81E-56
78789	Vsig1	-5.95744	1.19E-05	3.39E-05
237310	Il22ra2	-5.89815	4.89E-05	0.000131
14317	Ftcd	-5.87517	1.57E-32	1.50E-31
268958	Capn11	-5.83692	4.89E-05	0.000131
12889	Cplx1	-5.70496	0.000409	0.000984
13166	Dbh	-5.66327	0.000409	0.000984
331374	Dgkk	-5.65699	2.24E-14	1.12E-13
15558	Htr2a	-5.59687	8.87E-14	4.30E-13
56485	Slc2a5	-5.46417	9.93E-05	0.000257
241520	Fam171b	-5.4385	4.89E-05	0.000131
208188	Ghsr	-5.41578	2.88E-06	8.67E-06
17885	Myh8	-5.36421	1.08E-11	4.72E-11
76574	Mfsd2a	-5.29021	3.45E-21	2.35E-20
102075	Plekhg4	-5.14248	4.89E-05	0.000131
20681	Sox8	-5.08668	1.29E-09	4.96E-09
15116	Has1	-5.08668	1.29E-09	4.95E-09
13371	Dio2	-5.06465	6.18E-18	3.67E-17
66198	Them5	-5.06465	6.18E-18	3.67E-17
246317	Neto1	-5.03349	0.000409	0.000984

**Figure 2:**
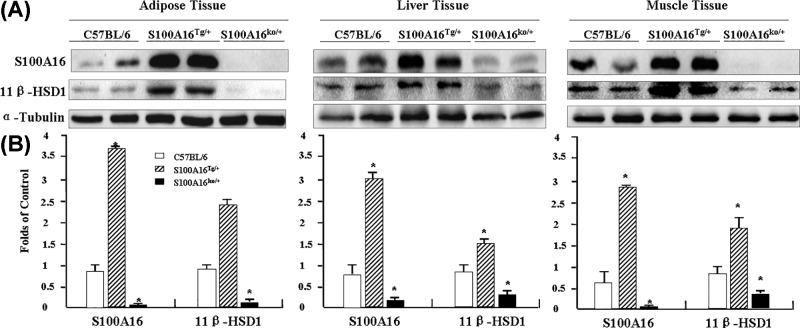
Effect of S100A16 on 11β-HSD1 expression Adipose, liver, and muscle tissues were removed from C57BL/6, S100A16^Tg/Tg^, and S100A16^KO/+^ mice, and proteins were extracted. (**A**) Analysis of S100A16 and 11β-HSD1 protein levels by Western blotting. (**B**) Relative expression of S100A16 and 11β-HSD1 based on grayscale analysis with α-tubulin as a control.

We did not observe any changes in the mRNA expression of 11β-HSD1. However, when we compared 11β-HSD1 protein levels in S100A16^Tg/Tg^ and S100A16^KO/+^ mice with those in C57BL/6 mice using Western blotting, 11β-HSD1 was up-regulated in S100A16^Tg/Tg^ mice and down-regulated in S100A16^KO/+^ mice in all these three tissues (visceral adipose, liver, and skeletal muscle from the hind legs) (Figure 2A,B). This difference in S100A16 expression further confirmed that S100A16^Tg/Tg^ and S100A16^KO/+^ mice had been engineered successfully.

### S100A16 and 11β-HSD1 increased lipid droplets in 3T3-L1cells

**Figure 3:**
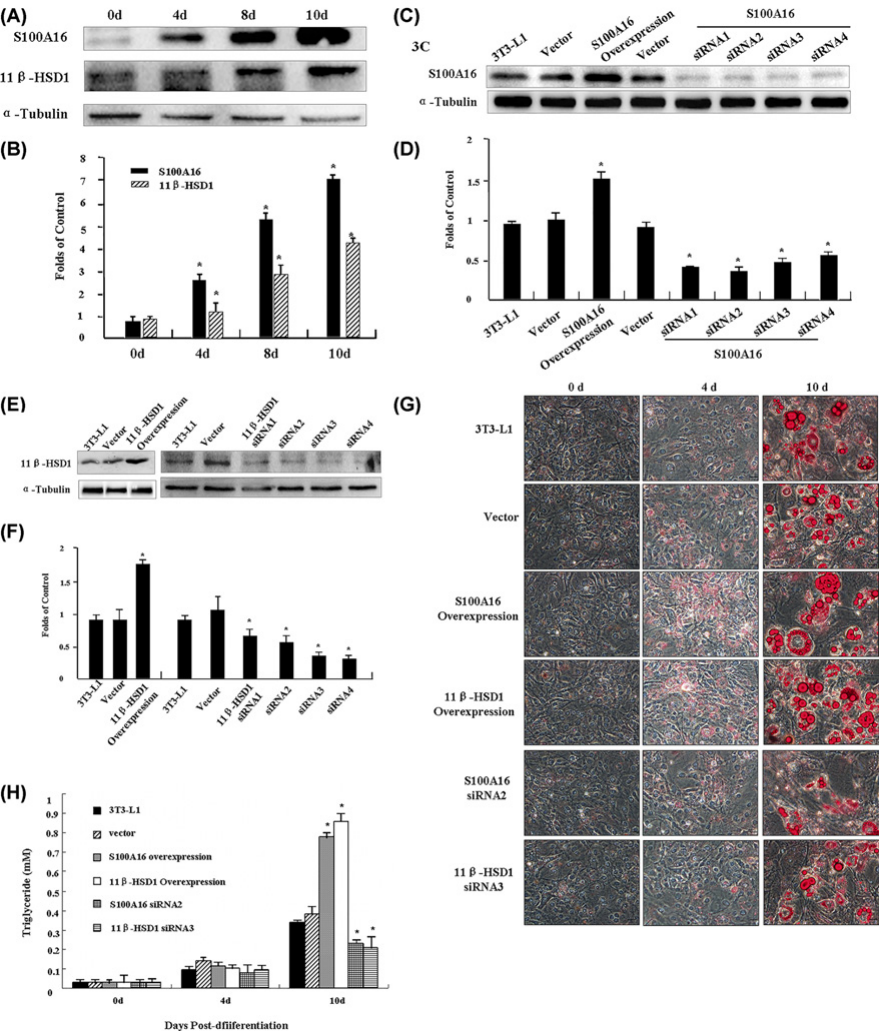
Effects of S100A16 and 11β-HSD1 on preadipocyte differentiation (**A**) Analysis of S100A16 and 11β-HSD1 protein levels by Western blotting in protein extracts collected at 0, 4, 8, and 10 days after induction of differentiation with α-tubulin as a control. (**B**) Quantification of S100A16 and 11β-HSD1 expression described in (**A**) based on grayscale analysis. (**C**) Western blotting of S100A16 protein levels in various transfectants. (**D**) Quantification of S100A16 expression described in (**C**) based on grayscale analysis. (**E**) Western blotting analysis of 11β-HSD1 protein levels in various transfectants. (**F**) Quantification of 11β-HSD1 expression described in (**E**) based on grayscale analysis. (**G**) Induction of differentiation of 3T3-L1 transfectants overexpressing 11β-HSD1 or S100A16 into adipocytes followed by fixing and staining with oil red O at different time points (0, 4, and 10 days). And induction of differentiation of 3T3-L1 transfectants in which 11β-HSD1 or S100A16 were down-regulated into adipocytes followed by fixing and staining with Oil Red O at different time points (0, 4, and 10 days). Photographs were taken using a light microscope at 200× magnification (LEICA). *P* ≤0.05 compared to controls. (**H**) Quantitative determination of triglyceride accumulation in cells. The lipid levels were determined using a Triglyceride GPO-POD assay kit. Results were expressed as the mean±SD for *n* = 3. *P* ≤ 0.05 when compared with the corresponding results in the control cell lines.

Expression of S100A16 and 11β-HSD1 at the protein level was measured by Western blotting while 3T3-L1 preadipocytes differentiated into adipocytes. Upon induction of differentiation into adipocytes, expression of both S100A16 and 11β-HSD1 was significantly elevated, by 7.2- and 4.5-fold at 10 days post-differentiation (Figure 3A,B). To assist our understanding of the functional roles of S100A16 and 11β-HSD1 in differentiation, we altered the expression levels in 3T3-L1 preadipocytes using the pcDNA3.1 overexpression system or by transfection with pcDNA6.2-GW/ EmGFP-miR (see ‘Materials and Methods’ section). In either case, we established S100A16-overexpressing and -deficient cell lines and 11β-HSD1-overexpressing and -deficient cell lines for analysis of differentiation (Figure 3C,F).

We investigated whether elevated S100A16 or 11β-HSD1 expression promotes adipogenesis in 3T3-L1 cells harboring different expression vectors by plating them in high density (near confluence) and allowing them to grow for 48 h before initiating differentiation (see ‘Materials and Methods’ section). At different time points after differentiation, Oil Red O staining was applied and microscopy performed to detect cellular lipid droplets. We found that overexpression of S100A16 or 11β-HSD1 led to a marked increase in Oil Red O staining, beginning as early as 10 days after the start of the observation period (Figure 3G), and a reduction in either S100A16 expression (via siRNA2) or 11β-HSD1 expression (via siRNA3) decreased the Oil Red O staining (Figure 3G). Consistent with such staining patterns, quantitative analysis of cellular triglycerides showed that triglyceride accumulation was significantly higher in the cells overexpression of S100A16 and 11β-HSD1 but lower in the cells expression of specific S100A16 siRNA2 and 11β-HSD1 siRNA3, than the control cells (Figure 3H). These results suggest that both S100A16 and 11β-HSD1 promote 3T3-L1 differentiation into adipocytes.

### Effects of S100A16 on 11β-HSD1 activity

**Figure 4:**
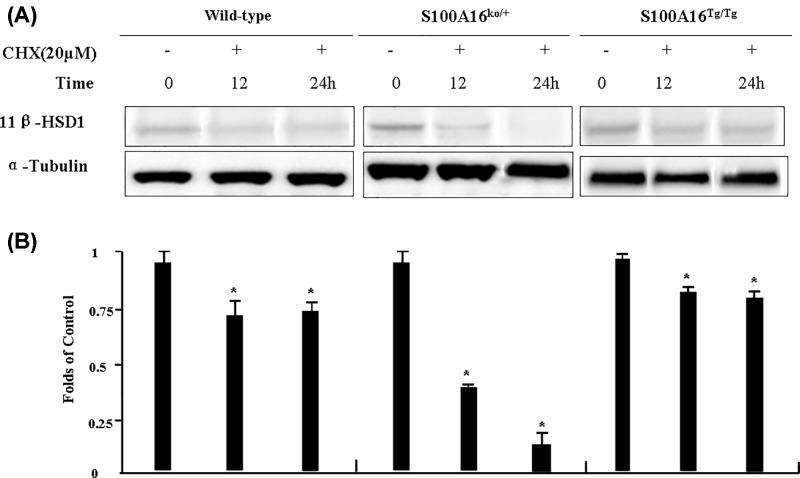
Effect of S100A16 on 11β-HSD1 activity (**A**) Mouse embryonic fibroblasts (MEFs) from C57BL/6, S100A16^Tg/Tg^ and S100A16^KO/+^ mice were treated with cycloheximide (CHX) at 20 μM for different times (0, 12, or 24 h) and cell lysates were subjected to immunoblotting using anti-11β-HSD1 antibody. (**B**) Quantification of 11β-HSD1 expression described in (A) based on grayscale analysis. *P≤0.05 compared to controls.

The above results showed that both S100A16 and 11β-HSD1 have an inductive effect on preadipocyte differentiation, and importantly, S100A16 has a positive influence on 11β-HSD1 protein expression, but the mechanisms remained unknown. We therefore examined numerous molecular signals but the results were not conclusive (data not show). Therefore, we want to investigate whether the stabilization of 11β-HSD1 was affected by S100A16 protein in MEF cells. To study the effect of S100A16 on 11β-HSD1 protein degradation, 20 μM cycloheximide (CHX) was used as a protein synthesis inhibitor. The Western blotting results showed that overexpression of S100A16 inhibited the degradation of 11β-HSD1, and down-regulation of S100A16 had the opposite effect (Figure 4).

## Discussion

Our main aims in the present study were (1) to better understand the effect of S100A16 on fat metabolism and insulin resistance, and (2) to gain insight into the mechanism by which S100A16 regulates 11β-HSD1 in adipose tissue. Using a transgenic mouse model fed a NFD or HFD, we investigated the effect of S100A16 on weight gain, visceral fat, insulin sensitivity, and 11β-HSD1 expression. Additionally, using the 3T3-L1 cell model, we investigated the effect of S100A16 and 11β-HSD1 on pre-adipocyte differentiation, and using MEF cells, we investigated the mechanism by which S100A16 up-regulates 11β-HSD1 expression. We showed that overexpression of S100A16 enhanced body weight in HFD mice, and that S100A16 up-regulates 11β-HSD1 protein levels by inhibiting its degradation. These findings are consistent with the ability of 11β-HSD1 to promote preadipocyte differentiation.

S100A16 is a calcium-binding protein that is ubiquitously expressed in human tissues and highly expressed in a variety of tumors [20,21]. Thus, along with many other S100 family members, it may be a potential marker of tumor cells and is likely involved in the regulation of cell growth. However, perturbation of cellular S100A16 resulted in only a modest effect on the proliferation of 3T3-L1 preadipocytes, although it did have a profound impact on differentiation into adipocytes [22,4]. In this context, our present *in vivo* and *in vitro* results significantly advance our knowledge by showing that S100A16^Tg/Tg^ mice gradually developed a significantly higher body weight than C57BL/6 mice, and the weight of their visceral fat pads consistently exceeded those of the C57BL/6 group (Figure 1B). Additionally, overexpression of S100A16 led to a marked increase in lipid droplet accumulation as early as 10 days after the start of the observation period while down-regulation of S100A16 expression by siRNA2 had the reverse effect (Figure 3G). RNAseq was done, but no pro-adipogenic markers were identified, which raised the possibility that adipogenesis itself has not been promoted and lipid homeostasis might be disrupted. And this idea need more experiments in futhure.

Obesity is known to increase the risk of metabolic diseases. Increased lipid accumulation in ectopic tissues, including skeletal muscle and liver, is associated with the pathogenesis of non-alcoholic fatty liver disease, cardiac dysfunction, heart failure, and impaired insulin signaling [23–25]. Consistent with this, we showed that overexpression of S100A16 induced lipid accumulation in adipose and liver tissue (Figure 1G), and triggered insulin resistance (Figure 1C,D). Using the intraperitoneal glucose tolerance test, we found that S100A16^Tg/+^ mice fed a HFD displayed impaired glucose tolerance. Furthermore, consistent with the observed glucose intolerance, the response in blood glucose concentration to an intraperitoneal injection of insulin was also impaired.

11-β-hydroxysteroid dehydrogenase 1 (11β-HSD1) converts cortisone to cortisol, and this enzyme is ubiquitously distributed in glucocorticoid target organs [26]. Up-regulation of 11β-HSD1 is followed by adult obesity and metabolic syndrome, and 11β-HSD1 levels increase with differentiation of mature adipocytes [27]. Therefore, when we found that S100A16^Tg/+^ mice gradually developed significantly higher body weight than C57BL/6 mice, we decided to measure 11β-HSD1 expression, and found it to be up-regulated in S100A16^Tg/Tg^ mice and down-regulated in S100A16^KO/+^ mice in fat, liver, and muscle tissue (Figure 2A,B). Additionally, both S100A16 and 11β-HSD1 promoted 3T3-L1 differentiation into adipocytes (Figure 3). These results indicated that S100A16 may be associated with 11β-HSD1. We therefore investigated the effect of S100A16 on gene expression using RNA seq. mRNAs are differentially abundant in C57BL/6 and S100A16^Tg/Tg^ mice. The results revealed that many genes expression were up- or down-regulated at least 6-fold in the adipose tissue of S100A16^Tg/Tg^ mice (Table 1), while other genes expression were altered more than 5-fold between C57BL/6 and S100A16^KO/+^ mice (Table 2). We did not observe any changes in 11β-HSD1 mRNA levels. These results suggest that the increase in 11β-HSD1 protein levels was due, at least in part, to stabilization against protein degradation. We did not exclude the effect of S100A16 overexpression in other tissues. The phenotype may be a result of gene over expression in the brain to generate an impact on energy balance.

CHX, a well-known inhibitor of protein biosynthesis in eukaryotic cells, binds to the ribosomal E-site and inhibits eukaryotic elongation factor 2 (eEF2)-mediated tRNA translocation [28]. We used 20 μM CHX as a protein synthesis inhibitor to study the effect of S100A16 on 11β-HSD1 protein degradation in MEF cells. The Western blotting results showed that overexpression of S100A16 inhibited the degradation of 11β-HSD1, while down-regulation of S100A16 had the reverse effect (Figure 4). Although the underlying mechanisms remain to be systematically investigated, the initial results described here offer some mechanistic insight into how S100A16 expression impacts 11β-HSD1 activity. There may not be opposite effect between knockdown and trangene of S100A16. Both move in a similar direction however since the tansgene is under the CAG promoter protein expression is no longer regulated by endogenous mechanisms, hence degradation does not occur to a similar extent. Whereas for the knockdown of S100A16, CHX has inhibited protein synthesis to a greater extent. We need more experiments to validate this view.

## Conclusions

In conclusion, we showed that overexpression of S100A16 induced preadipocyte differentiation and stimulated body weight gain in mice fed a high fat diet via a mechanism that involves 11β-HSD1.

## Supporting information

**Supplementary Figure S1:** Genotyping of S100A16 transgenic (S100A16^Tg/+^) mouse using PCR.

**Supplementary Figure S2:** The levels of S100A16 isoforms in wild-type C57BL/6 and S100A16 transgenic heterzygous (S100A16^Tg/+^) mice were determined by Q-PCR.

**Supplementary Figure S3:** Targeted Disruption of the s100a16 Locus.

**Supplementary Table S1:** Product Data-D12451

**Supplementary Table S2:** S100A16 Reverse Complementary Oligonucleotides

**Supplementary Table S3:** 11β-HSD1 Reverse Complementary Oligonucleotides

## Abbreviations

11β-HSD1: 11-β-hydroxysteroid dehydrogenase 1
HFD: high fat diet
NFD: normal fat diet
S16^KO/+^: S100A16 knockout heterozygous mouse
S16^Tg/+^: S100A16 transgenic heterozygous mouse
