# Supplementary material for: S100A16-induced adipogenesis is associated with up-regulation of 11 β-hydroxysteroid dehydrogenase type 1 (11β-HSD1)
Source: Biosci Rep. 2019 Sep 9;39(9):BSR20182042. doi: 10.1042/BSR20182042 (PMC6734118; doi:10.1042/BSR20182042)
Supplement: Supplementary file 1 [file bsr20182042_Supp1.pdf]

Supplementary Table1. Product Data-D12451

Formula

Product #D12451		gm%	kcal%
Protein		24	20
Carbohydrate		41	35
Fat		24	45
	Total		100
	kcal/gm	4.73	

Ingredient	gm	kcal
Casein, 30 Mesh	200	800
L-Cystine	3	12
Corn Starch	72.8	291
Maltodextrin 10	100	400
Sucrose	172.8	691
Cellulose, BW200	50	0
Soybean Oil	25	225
Lard*	177.5	1598
Mineral Mix S10026	10	0
DiCalcium Phosphate	13	0
Calcium Carbonate	5.5	0
Potassium Citrate, 1 H2O	16.5	0
Vitamin Mix V10001	10	40
Choline Bitartrate	2	0
FD&C Red Dye #40	0.05	0
**Total**	**858.15**	**4057**

Formulated by E. A. Ulman, Ph.D., Research Diets, Inc., 8/26/98 and 3/11/99.

*Typical analysis of cholesterol in lard = 0.72 mg/gram.
Cholesterol (mg)/4057 kcal = 167.8
Cholesterol (mg)/kg = 195.5

Supplementary Table 2. S100A16 Reverse Complementary Oligonucleotides

oligo DNA	5’-3’
13MR0109-01-F	TGCTGAACAACTGCCTTCTCCAGCTCGTTTTGGCCACTGACTGACGAGCTGGAAGGCAGTTG[illegible]
13MR0109-01-R	CCTGAACAACTGCCTTCCAGCTCGTCAGTCAGTGGCCAAAACGAGCTGGAGAAGGCAGTTG[illegible]
13MR0109-02-F	TGCTGTTCTGGATGAGCTTGTCAGCTGTTTTGGCCACTGACTGACAGCTGACACTCATCCAG[illegible]
13MR0109-02-R	CCTGTTCTGGATGAGTGTCAGCTGTCAGTCAGTGGCCAAAACAGCTGACAAGCTCATCCAG[illegible]
13MR0109-03-F	TGCTGTTCGGAAGCTGGACTTGCTGAGTTTTGGCCACTGACTGACTCAGCAAGCAGCTTCCG[illegible]
13MR0109-03-R	CCTGTTCGGAAGCTGCTTGCTGAGTCAGTCAGTGGCCAAAACTCAGCAAGTCCAGCTTCCGA[illegible]
13MR0109-04-F	TGCTGTGGTGATTCCACCAATCATGGGTTTTGGCCACTGACTGACCCATGATTTGGAATCAC[illegible]
13MR0109-04-R	CCTGTGGTGATTCCAAATCATGGGTCAGTCAGTGGCCAAAACCCATGATTGGTGGAATCAC[illegible]
Negative-F	TGCTGAAATGTACTGCGCGTGGAGACGTTTTGGCCACTGACTGACGTCTCCACGCAGTACAT[illegible]
Negative-R	CCTGAAATGTACTGCGTGGAGACGTCAGTCAGTGGCCAAAACGTCTCCACGCGCAGTACAT[illegible]

Supplementary Table 3. 11β-HSD1 Reverse Complementary Oligonucleotides

Oligo DNA	5'-3'
14MR0018-1-F	TGCTGTTTCCTTCATAGCTGTTTCTGGTTTTGGCCACTGACTGACCAGAAACATATGAAGGAAA
14MR0018-1-R	CCTGTTTCCTTCATATGTTTCTGGTCAGTCAGTGGCCAAAACCAGAAACAGCTATGAAGGAAAC
14MR0018-2-F	TGCTGTAAATTGCTCCGCAAATGTCAGTTTTGGCCACTGACTGACTGACATTTGGAGCAATTTA
14MR0018-2-R	CCTGTAAATTGCTCCAAATGTCAGTCAGTCAGTGGCCAAAACTGACATTTGCGGAGCAATTTAC
14MR0018-3-F	TGCTGAGGCCAAGGACACAGAGAGTGGTTTTGGCCACTGACTGACCACTCTCTGTCCTTGGCCT
14MR0018-3-R	CCTGAGGCCAAGGACAGAGAGTGGTCAGTCAGTGGCCAAAACCACTCTCTGTGTCCTTGGCCTC
14MR0018-4-F	TGCTGATGATATGCCATTTCTCTTCCGTTTTGGCCACTGACTGACGGAAGAGATGGCATATCAT
14MR0018-4-R	CCTGATGATATGCCATCTCTTCCGTCAGTCAGTGGCCAAAACGGAAGAGAAATGGCATATCATC
Negative-F	TGCTGAAATGTACTGCGCGTGGAGACGTTTTGGCCACTGACTGACGTCTCCACGCAGTACATTT
Negative-R	CCTGAAATGTACTGCGTGGAGACGTCAGTCAGTGGCCAAAACGTCTCCACGCGCAGTACATTTC

Supplementary Fig. 1

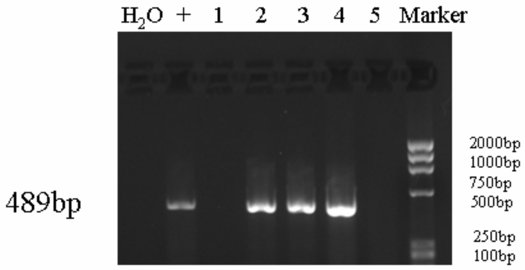

H_2O: negtive control, +: postive control, 1:C57BL/6, 2:S100A16$^{Tg/+}$, 3:S100A16$^{Tg/+}$, 4:S100A16$^{Tg/+}$, 5:S100A16$^{Tg/+}$,

Pimer : S100A16$^{Tg/+}$-F 5'-TTGTGCTGTCTCATCATTTTGG-3'
S100A16$^{Tg/+}$-R 5'-ACCAGCCACCACCTTCTGATA-3'

Supplementary Fig.1. Genotyping of S100A16 transgenic (S100A16$^{Tg/+}$) mouse using PCR.

Total RNA from tails of C57BL/6 and S100A16$^{Tg/+}$ mouse was purified using Trizol Reagent (Invitrogen). The allele S100A16 was identified using PCR, and S100A16 region as shown in 489bp line in this figure. H_2O: negtive control, +: postive control, 1:C57BL/6, 2:S100A16$^{Tg/+}$, 3:S100A16$^{Tg/+}$, 4:S100A16$^{Tg/+}$, 5:S100A16$^{Tg/+}$, Marker: 100bp, 250bp, 500bp, 750bp, 1000bp,2000bp.

Supplementary Fig. 2

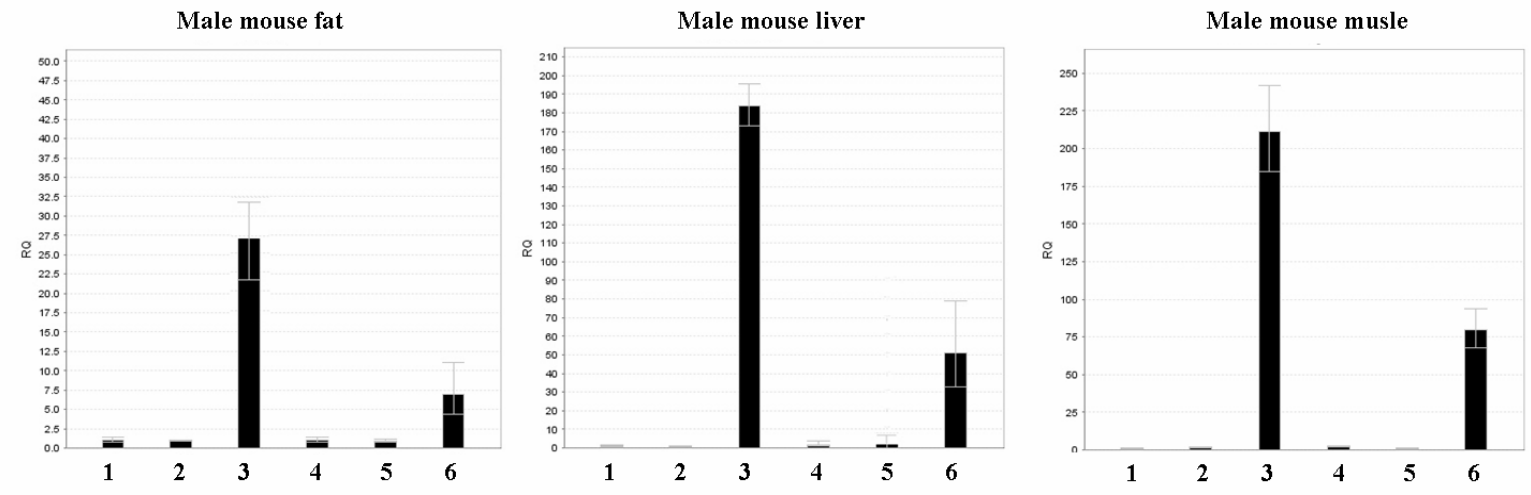

1:control 1, 2: control 2, 3:S100A16$^{Tg/Tg}$, 4:C57BL/6, 5:C57BL/6, 6:S100A16$^{Tg/+}$

Supplementary Fig.2. The levels of S100A16 isoforms in wild-type C57BL/6 and S100A16 transgenic heterzygous (S100A16$^{Tg/+}$) mice were determined by Q-PCR.

Total RNAs from fat, liver, and musle of the male mice were purified using Trizol Reagent. The primers are β-actin-F 5'-TGACGTGGACATCCGCAAAG-3', β-actin-R 5'-CTGGAAGGTGGACAGCGAGG-3';S100A16-F5'-AGTACAGCCTGGTCAAGA ACA-3', S100A16-R 5'-TCCCTGTGTCCGACAGCAT-3'. For the Q-PCR reaction, the total RNA was mixed with the primers according to the manufacturer's instructions of the SYBR Premix Ex Taq (TAKARA RR420A).

Supplementary Fig. 3

A Targeted disruption of the S100A16 locus in mice

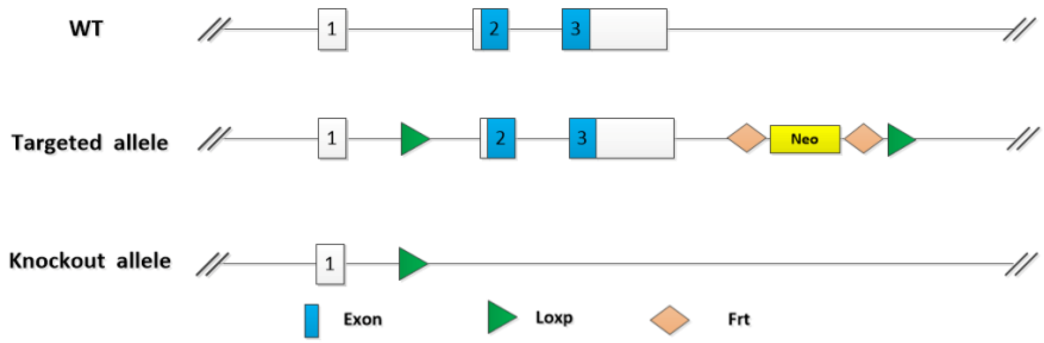

B Genotyping primers

Primer 1 S100A16$^{KO/+}$-1F 5'-ACTACCTTAGAGCC TATGCAAGG-3',
S100A16$^{KO/+}$-1R 5'-GCTCCTGTGAGGAGAGGAAGC-3'
Wt:246bp; Flox:364bp

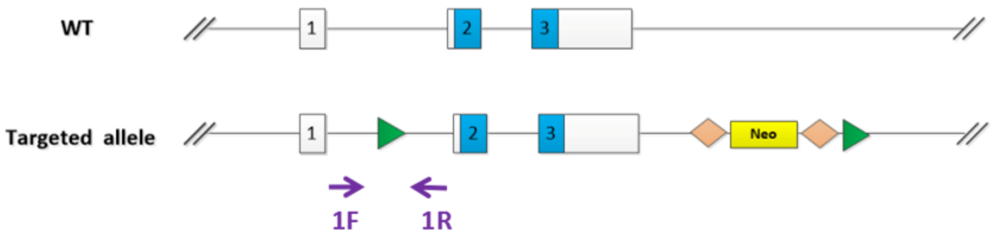

Primer 2 S100A16$^{KO/+}$-2F 5'-ACTACCTTAGAGCCTATGCAAGG-3',
S100A16$^{KO/+}$-2R 5'-GAGAGCTAGG AGGAGTGGATG-3' '
KO:388bp; Wt: no band (2.725 kb)

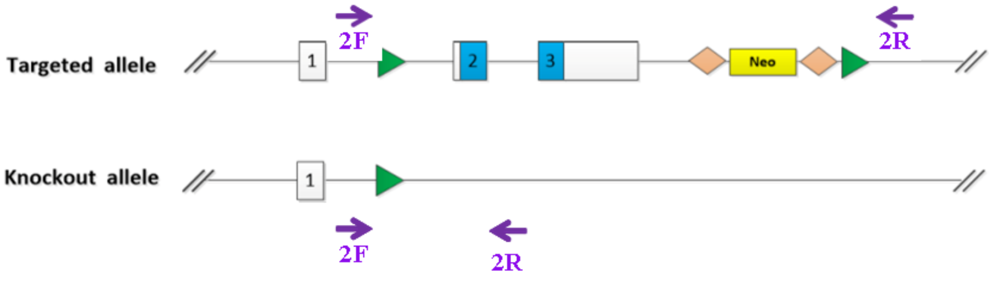

C PCR results

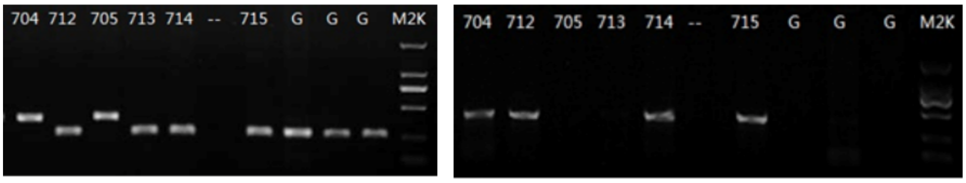

primer1: Wt: 246bp; Flox: 364bp primer2: KO: 388bp; Wt : no band（2.725kb）

G: genome --: H_2O M2K:DL2000:2000,1000,750,500,250,100

704#: S100A16$^{fl/KO}$
705#: S100A16$^{fl/fl}$
713#: S100A16$^{WT/WT}$
714#: S100A16$^{WT/ko}$

704#: S100A16$^{WT/KO}$
705#: WT
713#: WT
714#: S100A16$^{WT/ko}$

Supplementary Fig.3. Targeted Disruption of the s100a16 Locus.

S100A16 Knockout heterozygous mouse (S100A16$^{KO/+}$) were generated at the Model Animal Research Center Of Nanjing University. Loxp points were added before the second exon and behind the third exons in the N-terminal sequence of s100a16 gene (A). Embryonic stem cells with one of the s100a16 alleles deleted were used to create chimeras and further develop a stable heterozygous mouse strain. The mice were then back-crossed with C57BL/6 mice.The genotype of fetuses was determined by genomic PCR with specific primers (B). S100A16$^{KO/+}$-1F 5'-ACTACCTTAGAGCC TATGCAAGG-3', S100A16$^{KO/+}$-1R 5'-GCTCCTGTGAGGAGAGGAAGC-3'; S100A16$^{KO/+}$-2F 5'-ACTACCTTAGAGCCTATGCAAGG-3', S100A16$^{KO/+}$-2R 5'-GAGAGCTAGG AGGAGTGGATG-3'. (C), tails were cutted from the mice and total RNAs from tails were purified using Trizol Reagent. PCR was used to determine the genotypes of mice with specific primers.
